# Characterization of the complete chloroplast genome of *Canavalia gladiata*

**DOI:** 10.1080/23802359.2020.1861566

**Published:** 2021-01-27

**Authors:** Jun Zhao

**Affiliations:** YiBin Vocational and Technical College, Yibin, Sichuan

**Keywords:** *Canavalia gladiata*, *Canavalia*, chloroplast genome, Illumina sequencing

## Abstract

*Canavalia gladiata* is a member of genus *Canavalia*, which includes 60 species around world. And the species in genus of *Canavalia* have similar character so we use complete chloroplast genome to identify *C. gladiata* and other species. Here, we assembled and analyzed the complete chloroplast (cp) genome of *C. gladiata*. The cp genome of *C. gladiata* is 157,923 bp, with a total GC content of 34.6%. The complete cp genome has a typical quadripartite structure, including a large single-copy (LSC) region of 77,660 bp, a small single-copy (SSC) region of 18,945 bp and two inverted repeat (IR_S_) regions of 30,659 bp. The complete cp genome contains 136 genes, including 91 protein-coding genes, 37tRNA and 8 rRNA genes. Phylogenetic analysis result showed that *C. gladiata* is closely related to *C. rosea*.

*Canavalia gladiata* is a member of genus *Canavalia* in subfamily *Papilionoideae* and the genus *Canavalia* includes about 60 species around the world (Sauer 1964; Aymard and Cuello 1991). *Canavalia gladiata* is well known as originated in Asian, and distributed around Asia west Indian, Africa, South America and Australia (Herklots 1972; Kay 1979). *Canavalia gladiata* was used as food, Chinese herbal medicine and sauce of phytochemical and pharmaceutical products (Nanda et al. 1993; Eknayake et al. 1999; Arun et al. 2003). Nowadays, he chloroplast genome plays an important role in species identification by its characters such as uniparental inheritance, conserved sequence composition in coding regions and numerous variable sites (Bi et al. 2018). However, the published complete chloroplast genome were no more than 3 species in this genus and *C. gladiata* was not included. In this study, we sequenced and identified the complete chloroplast genome of *C. gladiata.*

We selected the sample in Yibin Botanical Garden (27°50′N, 103°36′E) on 1 August 2020. The voucher specimen (CG20208264ZJ) was deposited in Laboratory of Molecular Biology in YiBin Vocational and Technical College. We used the fresh leaves to extract DNA with the modified CTAB method (Doyle and Doyle 1987) and constructed the libraries with an average length of 350 bp using the NexteraXT DNA Library Preparation Kit (Illumina, San Diego, CA), then the libraries were sequenced on Illumina Novaseq 6000 platform, 2.81 Gb clean data was assembled by SPAdes v.3.11.0 software (Bankevich et al. 2012) using the chloroplast genome of *Canavalia rosea* (Accession number: LC554221) as the reference. Finally, the assembled complete cp genome was annotated by Plann software (Huang and Cronk 2015), and submitted to GenBank under the accession number of MT922037.

The total length of complete cp genome of *C. gladiata* is 157,923 bp, with a total GC content of 34.6%. The complete cp genome has a typical quadripartite structure, including a large single copy (LSC) region of 77,660 bp, a small single copy (SSC) region of 18,945 bp and two inverted repeat (IR_S_) regions of 30,659 bp. The complete cp genome contains 136 genes, including 91 protein coding genes, 37tRNA and. 8 rRNA genes. *trn*K-UUU*, rps*16*, trn*G-UCC*, atp*F*, rpo*C1*, trn*L-UAA*, trn*V-UAC*, pet*B*, pet*D*, rpl*16*, rpl*2*, ndh*B*, trn*I-GAU*, trn*A-UGC*, ndha* genes contained an intron, and *clp*P and *ycf*3 contained 2 introns.

To determine the phylogenetic position of *C. gladiata*, the complete chloroplast genome of *C. gladiata* was aligned with other 15 species in subfamily Papilionoideae from GenBank using Mafft-7.037 (Katoh and Standley 2013). Subsequently, the phylogenetic tree was constructed by IQTREE v1.6 (Nguyen et al. 2015; Hoang et al. 2018) with 1000 bootstraps replicates using Best-fit model. By using Crataegus kansuensis(NC_039374.1) as out group we got the result that *C. gladiata* was closely related to *C. rosea* (LC554221.1) (Figure 1). These information will provide useful genomic resources for the development of molecular markers and accurate identification of *C. gladiata*.

**Figure 1.**
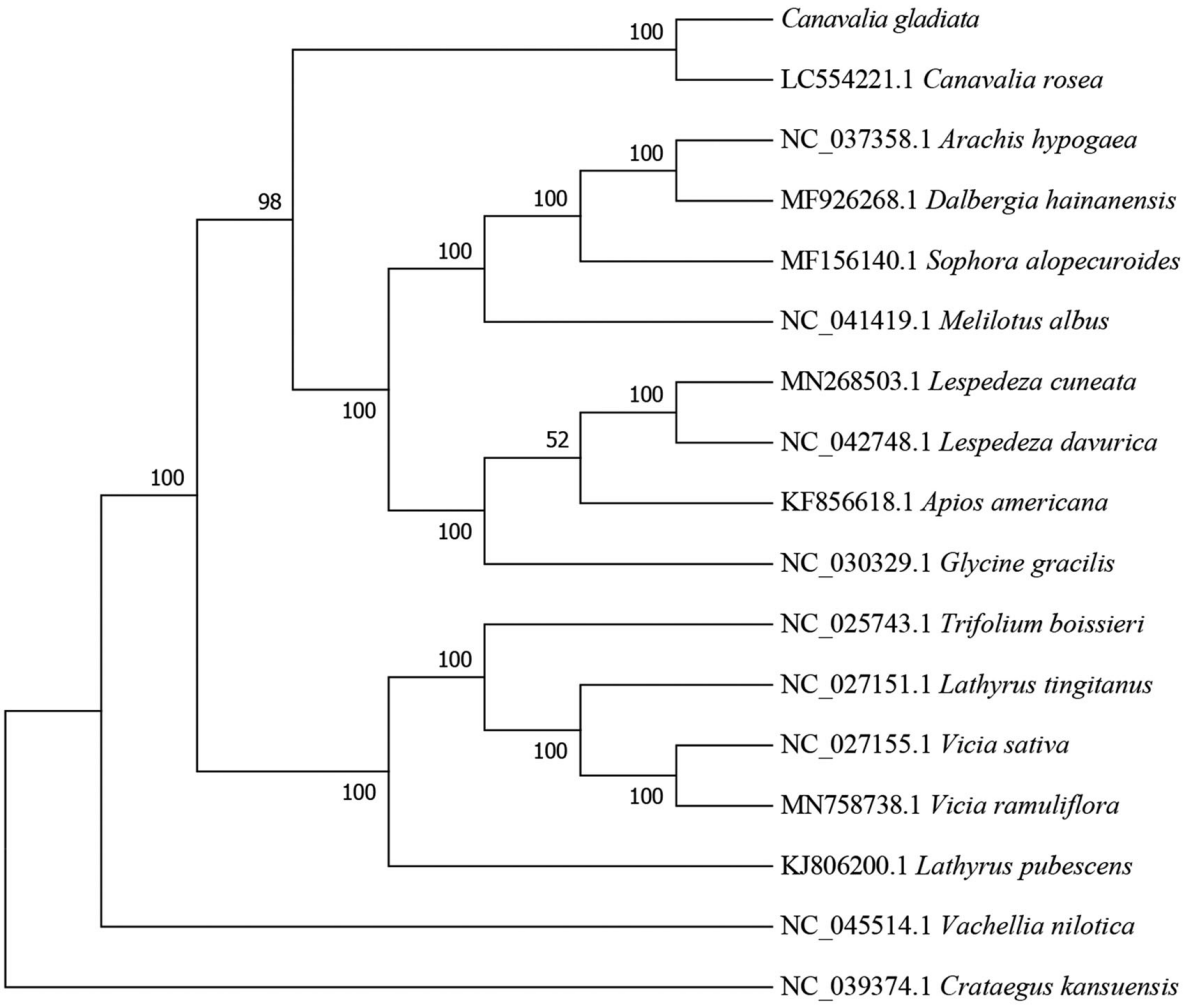
Maximum-likelihood phylogenetic tree for *Canavalia gladiata* based on 17 complete chloroplast genomes(*Canavalia rosea*, *Vachellia nilotica*, *Arachis hypogaea*, *Dalbergia hainanensis*, *Vicia sativa*, *Lathyrus tingitanus*, *Vicia ramuliflora*, *Lespedeza cuneata*, *Sophora alopecuroides*, *Glycine gracilis*, *Lathyrus pubescens*, *Trifolium boissieri*, *Apios americana*, *Lespedeza davurica*, *Melilotus albus*, *Canavalia gladiata* and *Crataegus kansuensis*). The Genbank accession numbers are on the diagram.

## Data Availability

The assembled complete chloroplast genome sequence of *Canavalia gladiata* has been submitted to GenBank under the accession number: MT922037 (https://www.ncbi.nlm.nih.gov/nuccore/MT922037).
